# Induction of renal artery hyperresponsiveness by alpha1-adrenoceptor in hepatorenal syndrome

**DOI:** 10.18632/oncotarget.22668

**Published:** 2017-11-25

**Authors:** Xiaogang Zhang, Xinsen Xu, Yina Jiang, Jianyu He, Wenjing Wang, Wei Li, Xufeng Zhang, Yi Lv

**Affiliations:** ^1^ Department of Hepatobiliary Surgery, First Affiliated Hospital of Xi'an Jiaotong University College of Medicine, Xi’an 710061, China; ^2^ Department of Pathology, First Affiliated Hospital of Xi'an Jiaotong University College of Medicine, Xi’an 710061, China; ^3^ Department of Pharmacology, First Affiliated Hospital of Xi'an Jiaotong University College of Medicine, Xi’an 710061, China

**Keywords:** hepatorenal syndrome, α1-adrenoceptor, hyperresponsiveness, renal artery, pathogenesis

## Abstract

**Objective:**

To investigate the potential role of alpha1-adrenoceptor (α1-AR) in the pathogenesis of hepatorenal syndrome.

**Methods:**

Hepatorenal syndrome was induced in male rats by intraperitoneal injection of D-galactosamine and orally treatment with α1-AR antagonist tamsulosin. Hyperresponsiveness of the renal artery contraction was evaluated by the laser-Doppler flowmetry and multimyograph system, while renal blood flow (cortical and medullary perfusion) was simultaneously measured. Renal artery ring segment tone was recorded with the myograph system, and concentration-response curves were obtained by cumulative administration of agonists.

**Results:**

This model developed acute renal and liver failure without renal damage in pathology, accompanied by significant hyperresponsiveness of renal artery contraction. After hepatorenal syndrome, plasma concentrations of tumor necrosis factor-α increased by two-fold, and α1-AR was significantly activated in the renal artery. Concentration-dependent vasoconstriction induced by noradrenaline was significantly decreased in the renal arteries of hepatorenal syndrome rat because of gradually decreased renal blood flow. Administration of tamsulosin prevented renal failure when given before the onset of liver injury, but it had no effect on liver injury by itself.

**Conclusion:**

α1-AR expression is positively associated with renal vasoconstriction induced by renal artery hyperresponsiveness in HRS. Therefore, α1-AR may be a potential target in the treatment of HRS.

## INTRODUCTION

Despite considerable advances in the treatment of end-stage liver disease, renal dysfunction remains common and contributes to the morbidity and mortality associated with hepatorenal syndrome (HRS) [1]. It was reported that renal blood flow was reduced in patients with severe acute liver failure (ALF), indicating renal vasoconstriction and pathological change in those patients who develop HRS [2]. However, the pathogenesis of ALF-induced acute renal failure (ARF) and its underlying molecular mechanisms are poorly understood, although several causal elements, such as splanchnic vasodilatation, reduction of effective arterial volume, and portal hypertension, have been considered as possible pathogenetic factors of renal injury [3, 4]. Therefore, events independent of systemic hemodynamics may be involved in the pathogenesis of HRS, including renal hemodynamic and/or renal vascular resistance changes. In HRS patients, splanchnic vasodilatation and decreased effective arterial volume antedate the development of renal failure, which also activates a variety of compensatory mechanisms (the renin-angiotensin system, sympathetic nervous system, and increased release of antidiuretic hormone etc.), leading to the renal vasoconstriction [3, 5]. As a result, the decrease of renal blood flow in HRS may be due to the overexpressed renovascular response induced vasoconstriction.

Although renal vasoconstriction is closely related to the development of ARF, it remains unknown about what kinds of receptor types are mainly and functionally responsible for detrimental effects in renovascular hyperresponsiveness [1, 6, 7]. It has been suggested that alpha1-adrenoceptor (α1-AR) is the functionally relevant adrenoceptor subtype in the renal vasculature of the rat. Additionally, α1-AR blockade has been reported to attenuate renal hemodynamic and functional changes induced by renal nerve stimulation and norepinephrine (NE) injection in the kidney [8]. Concurrent administration of α1-AR antagonists (prazosin and losartan) mitigated the fall in renal hemodynamics of ARF rats, although the effects of prazosin on renal function were not examined after ischemia reperfusion [9].

In this study, we characterized the renal dysfunction that occurs in the development of ALF induced by D-galactosamine (D-GalN) in an HRS rat model. We aimed to investigate the expression of the α1-AR subtype in renal vascular in this model, and to explore the effect of renal vasoconstriction induced by renovascular hyperresponsiveness via α1-AR modulation in HRS.

## RESULTS

### Characterization of a rat model of HRS

Preliminary experiments showed that approximately 16% of rats did not develop ARF (creatinine <50 μmol/L). The 48-hour mortality rate was 9%. This time point was chosen because the rats became unwell and the urine volume decreased at around 24-48 hours. This pattern was similar to that of humans, as about 50% of ALF patients in the intensive care unit develop acute renal dysfunction, with a 10-week mortality rate approaching 90% and median survival of around 1.7 weeks.

### Quantification of renal and hepatic function 48 hours after administration of D-GalN

Control (Group 1) and D-GalN (Group 2) rats had similar baseline renal and hepatic function. However, a dramatic increase was observed in the D-GalN (Group 2) rats over the controls, of the enzymes ALT (72.03 ± 16.77 vs. 5954.29 ± 473.80 IU/L; P < 0.01), AST (83.40 ± 13.39 vs. 6943.86 ± 411.70 IU/L; P < 0.01) and serum TBIL (1.13 ± 0.34 vs. 148.70 ± 33.48 μmol/L; P < 0.01) at 48 hours after administration of D-GalN (Table 1). The increase in ALT and AST was significantly higher in D-GalN (Group 2) rats with ARF. Serum albumin decreased significantly from 30.80 ± 3.82 to 26.30 ± 2.79 g/L (P < 0.05). As shown in Table 1, treatment of D-GalN rats with tamsulosin, either before (Group 4) or after (Group 5) administration of D-GalN, had no effect on the severity of liver injury at 48 hours.

**Table 1:** Serum biochemistry 48 hours after injection of D-galactosamine

Group	ALT (IU/L)	AST (IU/L)	TBIL (μmol/L)	Albumin (g/L)
Control (n = 10)	72.03 ± 16.77	83.40 ± 13.39	1.13 ± 0.34	30.80 ± 3.82
D-GalN (n = 10)	5954.29 ± 473.80 ^**^	6943.86 ± 411.70^**^	148.70 ± 33.48^**^	26.30 ± 2.79 ^*^
tamsulosin controls (n = 10)	74.90 ± 20.15	80.20 ± 12.99	1.06 ± 0.28	30.70 ± 3.40
tamsulosin pre-D-GalN (n = 10)	5877.32 ± 473.80 ^**^	7003.18 ± 383.25^**^	146.52 ± 27.58^**^	25.90 ± 2.65 ^*^
tamsulosin post-D-GalN (n = 10)	6020.46 ± 473.80 ^**^	6906.86 ± 353.67^**^	150.70 ± 31.94^**^	26.20 ± 2.28 ^*^

Values are mean ± SD.

^*^P < 0.05; ^**^P < 0.01, compared with control.

^*^P < 0.05; ^**^P < 0.01, compared with control.

Serum creatinine (40.86 ± 5.52 vs. 62.57 ± 5.19 μmol/L; P < 0.01) and BUN (4.97 ± 0.80 vs. 9.21 ± 1.09 mmol/L; P < 0.01) in the HRS rat model also increased significantly compared to control. Following D-GalN, a 53% decrease in Ccr (1.12 ± 0.14 vs. 0.52 ± 0.05 mL/min; P < 0.01), and an increase in urine creatinine (1899.7 ± 120.15 vs. 2732.86 ± 138.22 μmol/L; P < 0.01) were observed. Urinary sodium excretion decreased from 1.17 ± 0.11 to 0.79 ± 0.06 mmol/day (P < 0.05). However, FENa increased from 0.58% ± 0.03% to 1.73% ± 0.11% (P < 0.05), accompanied by a significant decrease in urine volume (15.89 ± 2.04 vs. 8.11 ± 1.55 mL/day) (P < 0.05). These data confirmed that renal function was also impaired in animals that developed ALF.

### Histology and quantification of hepatic and renal injury

**Figure 1:**
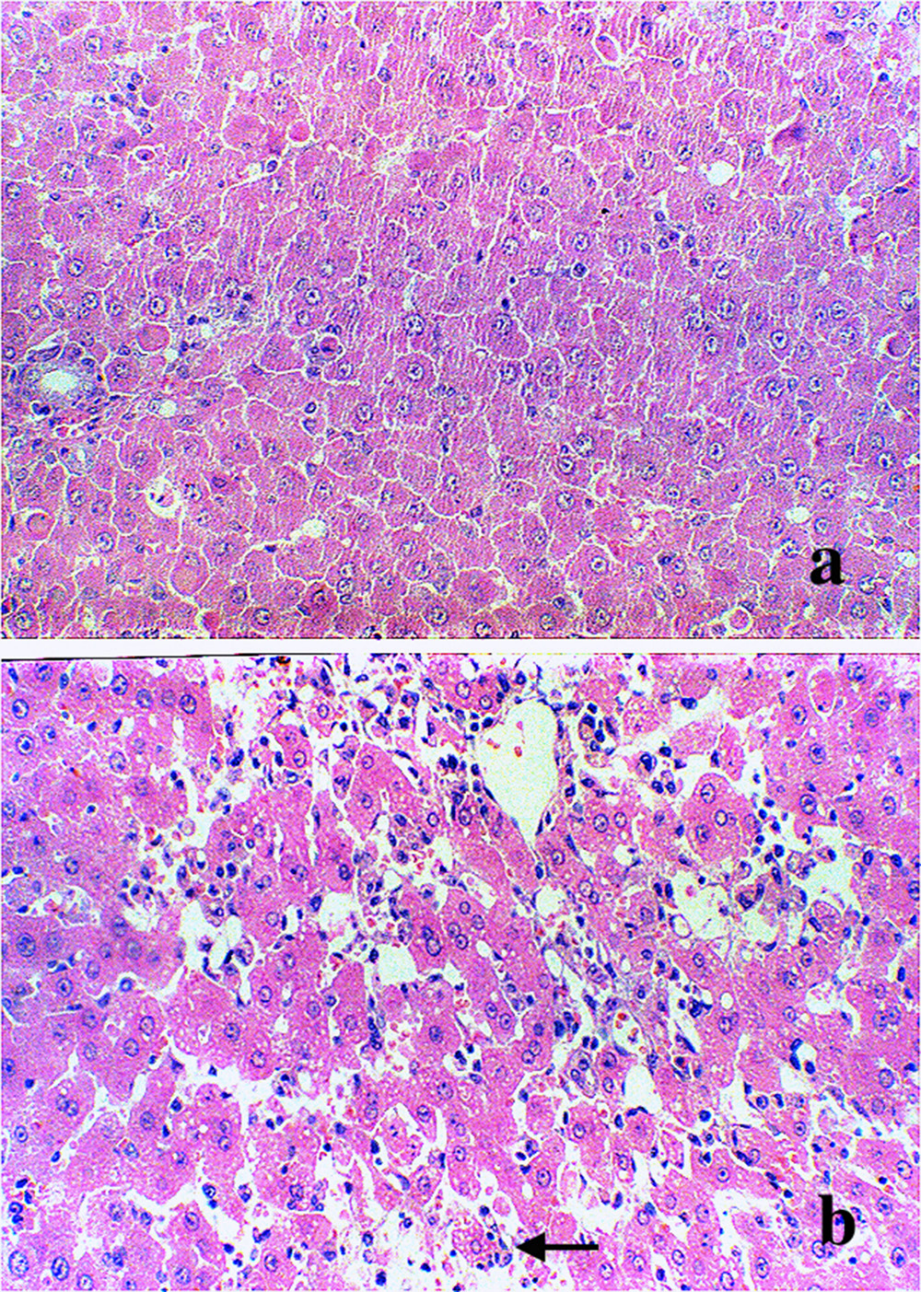
Morphological changes in the liver Light micrograph (original magnification × 200) of liver from **(a)** control rats and **(b)** 1200 mg/kg D-GalN injected rats at 48 hours. Hematoxylin and eosin stained section indicated dilatation of central vein and hepatic sinusoid. Vacuoles appeared in the cytoplasm of hepatocytes (black arrow). Hepatocellular necrosis could also be found in the D-GalN animal’s liver.

The microscopic examination of the livers of D-GalN (Group 2) rats revealed several pathologic changes. Histological evaluation indicated significant focal centrolobular necrosis of hepatocytes, disseminated manifestations of eosinophilic Councilman bodies, enlargement and proliferation of Kupffer cells, and focal accumulations of segmented leukocytes, lymphocytes, plasma cells, and histiocytes, all of these were not observed in the control rats (Figure 1).

**Figure 2:**
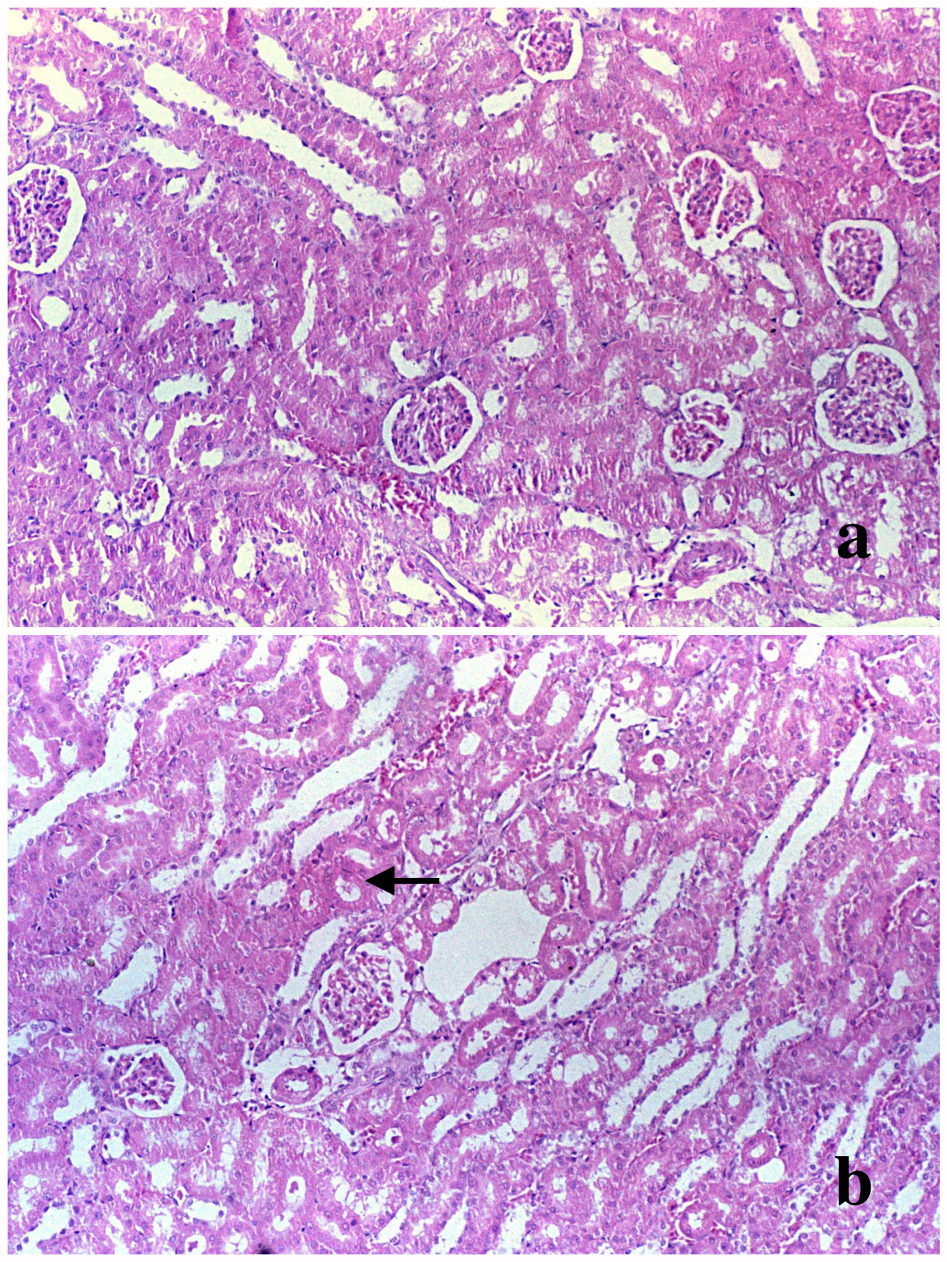
Morphological changes in the kidney Light micrograph (original magnification × 400) of kidney from **(a)** control rats and **(b)** 1200 mg/kg D-GalN injected rats at 48 hours. Hematoxylin and eosin stained section of kidneys showed no abnormality of the renal cortex or medulla in the D-GalN animals, except for slight thickening of the basement membranes of renal tubule (black arrow).

**Figure 3:**
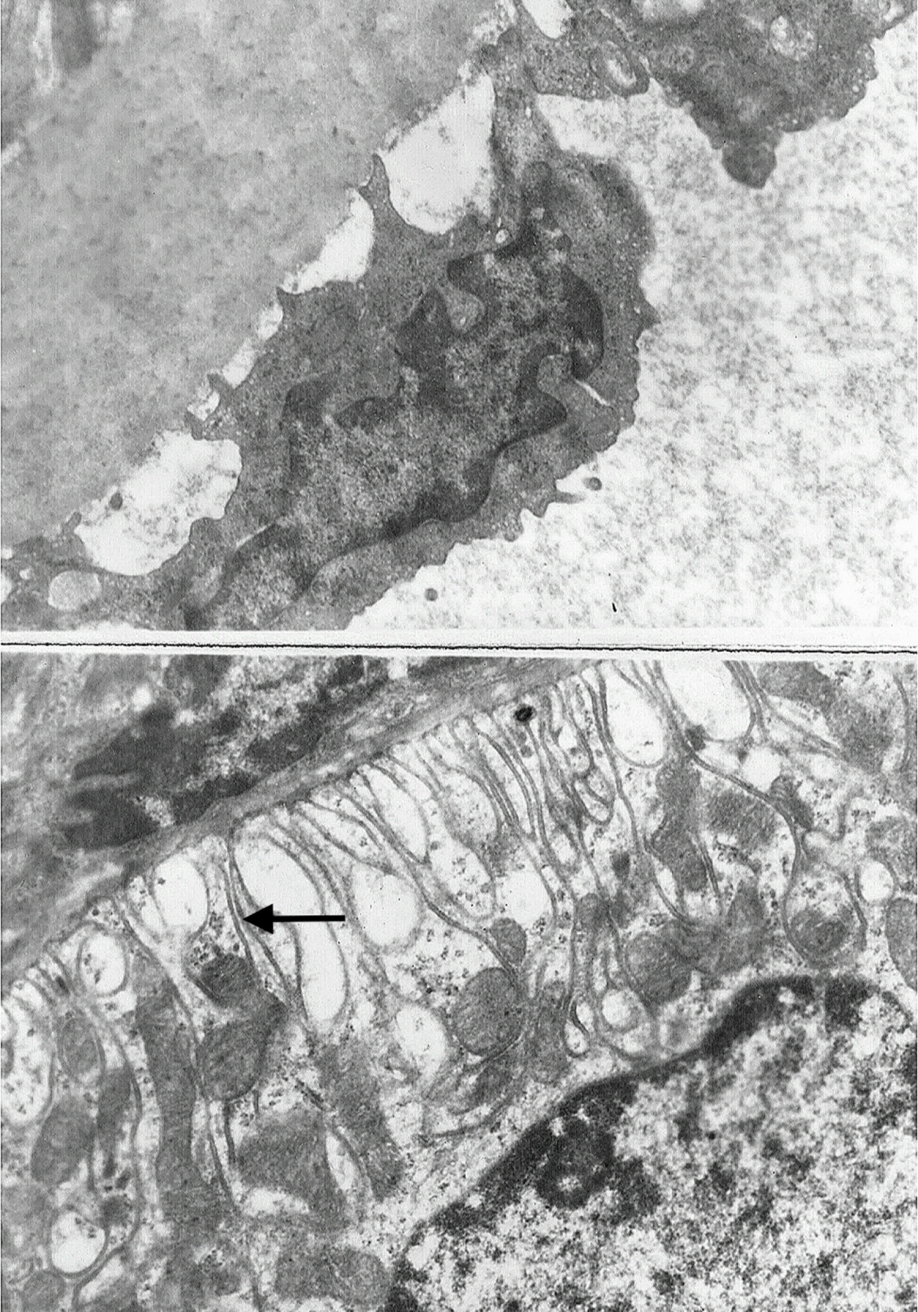
Electron micrograph (original magnification × 10000) of **(a)** renal artery and **(b)** kidney from 1200 mg/kg D-GalN-injected rats at 48 hours. Kidney and renal artery sections were fixed in glutaraldehyde, and stained with uracyl acetate and Reynold’s lead citrate. In D-GalN animals, the glomeruli and renal artery were normal. However, in the proximal tubules, the vacuolar system was slightly more prominent, with larger apical vacuoles as well as more prominent vacuoles, with flocculent proteinaceous material towards the base of the epithelial cells (black arrow).

Surprisingly, the kidney injury measured by the serum level of BUN and creatinine was not obviously displayed in renal histology in D-GalN (Group 2) rats compared to rats from control group. Except for slight thickening of the basement membranes of glomeruli, renal histopathological examinations at 48 hours did not show any severe lesions after D-GalN treatment in rats with ARF (Figure 2). Furthermore, examination of these kidneys by electron microscopy demonstrated almost normal glomeruli. However, the vacuolar system was slightly more prominent in the proximal tubules, with larger apical vacuoles and prominent vacuoles with flocculent proteinaceous material towards the base of the epithelial cells (Figure 3). In addition, histological changes characterized by minor focal lysis of disarranged smooth muscle cells in renal artery could still be observed in the D-GalN rats. The consequence of this increased prominence of the vacuolar system and focal lysis of smooth muscle cells in the renal artery of D-GalN rats is unknown.

### NE induced concentration-dependent vasoconstriction in renal arteries

**Figure 4:**
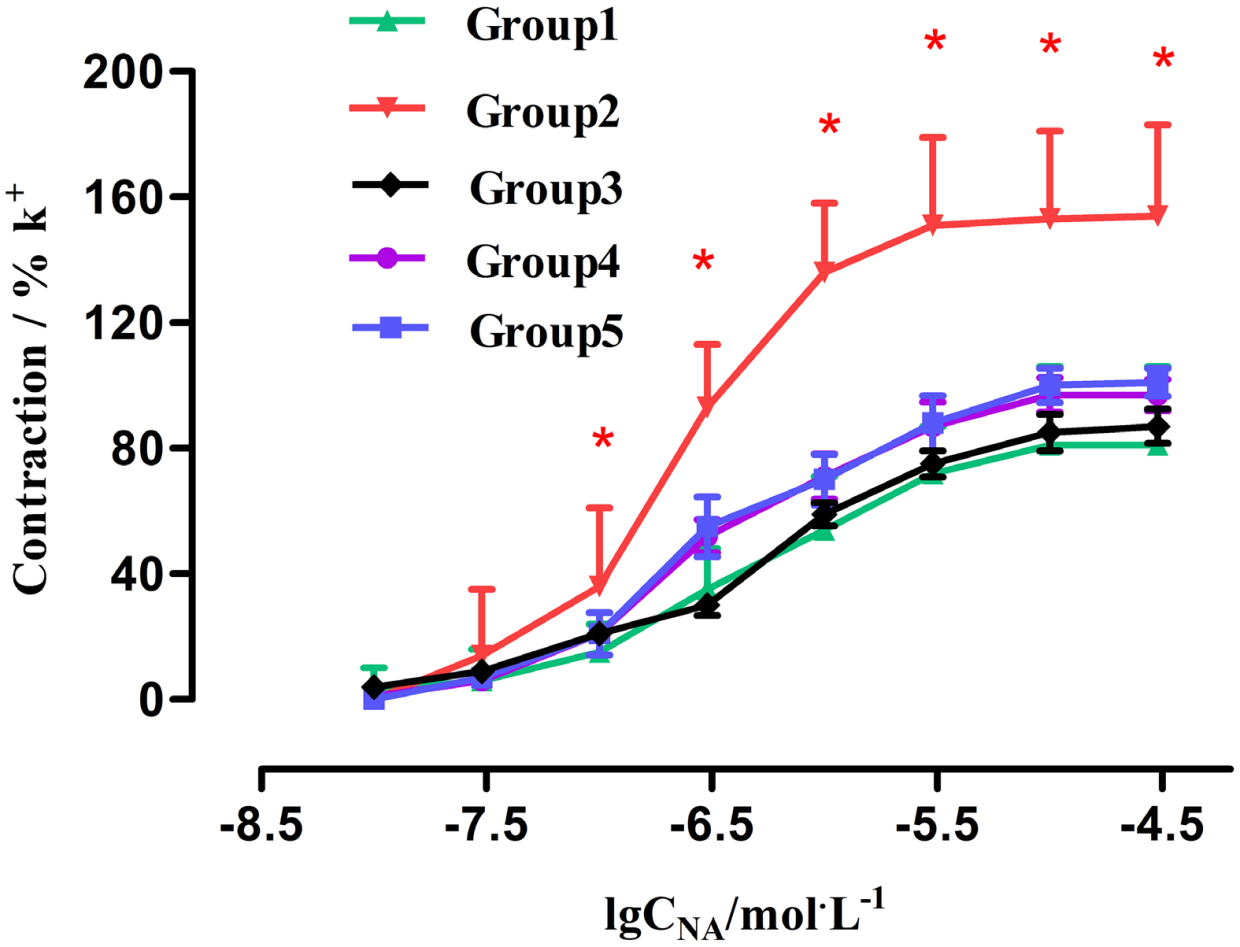
Effect of Tam on concentration-contractile curves induced by NE in rat renal artery Group1: control (n = 8); Group2: GalN (n = 8); Group4: Tam pre-D-GalN (n = 8); Group5: Tam post-D-GalN (n = 8). ^*^ P < 0.01, compared with control animals.

In Group 1, NE induced concentration-dependent vasoconstriction in rat renal arteries at a dose from 1 × 10^-8^ mol/L to 1 × 10^-4.5^ mol/L. The contractile effect occurred 20 seconds after agonist administration. We found that when reaching the maximal contraction, the response declined for about 2 minutes, and did not return to base line easily. E_max_ was 81% ± 25% and PD2 was 6.43 ± 0.39 in the NE-induced response curve for Group 1. The concentration-response curve is shown in Figure 4. In Group 2, NE induced contraction in renal arteries in a concentration-dependent manner at a dose from 1 × 10^-7.5^ mol/L^1^ to 1 × 10^-4.5^ mol/L^1^. The response took effect 10 seconds after addition of NE, and the contractile curve was significantly elevated. Furthermore, E_max_ and PD2 for this group significantly increased from 81% ± 25% to 154% ± 29% (P < 0.01), and from 6.43 ± 0.39 to 6.70 ± 0.18 (P < 0.05), respectively. The concentration-response curve was shown in Figure 4. The results showed a significant difference in NE-induced vasoconstriction in renal arteries between Groups 1 and 2. We found that after addition of D-GalN injection for 48 hours, NE-induced contraction in renal arteries was notably enhanced compared with the vessels of Group 1, suggesting functional upregulation of α1-AR in Group 2. This clearly indicated that arterial segments from HRS rats yielded an enhanced response to NE, compared with fresh vessel segments from control group rats.

### Interaction between renal cortical and medullary perfusion induced by upregulation of α1-AR

**Figure 5:**
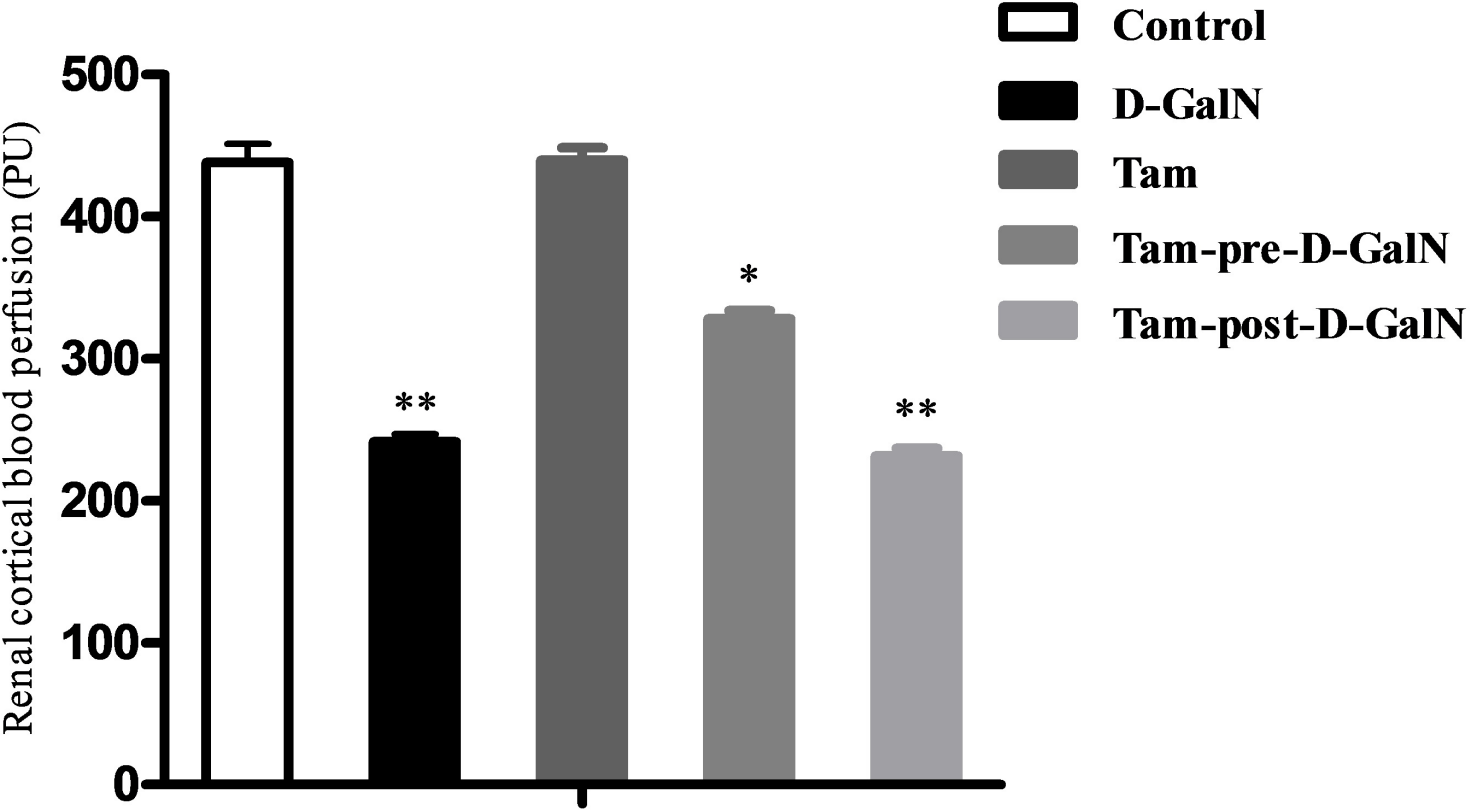
Assessment of renal cortical laser-Doppler flux (CLDF) in D-GalN rats and control rats Control: CLDF were measured at 48 hours after injection of saline. D-GalN: CLDF were measured at 48 hours after injection of D-GalN. Tam: CLDF were measured at 48 hours after administration of tamsulosin. Tam-pre-D-GalN: animals received tamsulosin 72 hours prior to D-GalN intraperitoneally, and CLDF were measured at 48 hours after administration of D-GalN. Tam-post-D-GalN: animals received tamsulosin 36 hours after D-GalN intraperitoneally, and CLDF were measured at 48 hours after administration of D-GalN. ^**^ P < 0.01, compared with control animals. ^*^ P < 0.01, compared with D-GalN animals.

**Figure 6:**
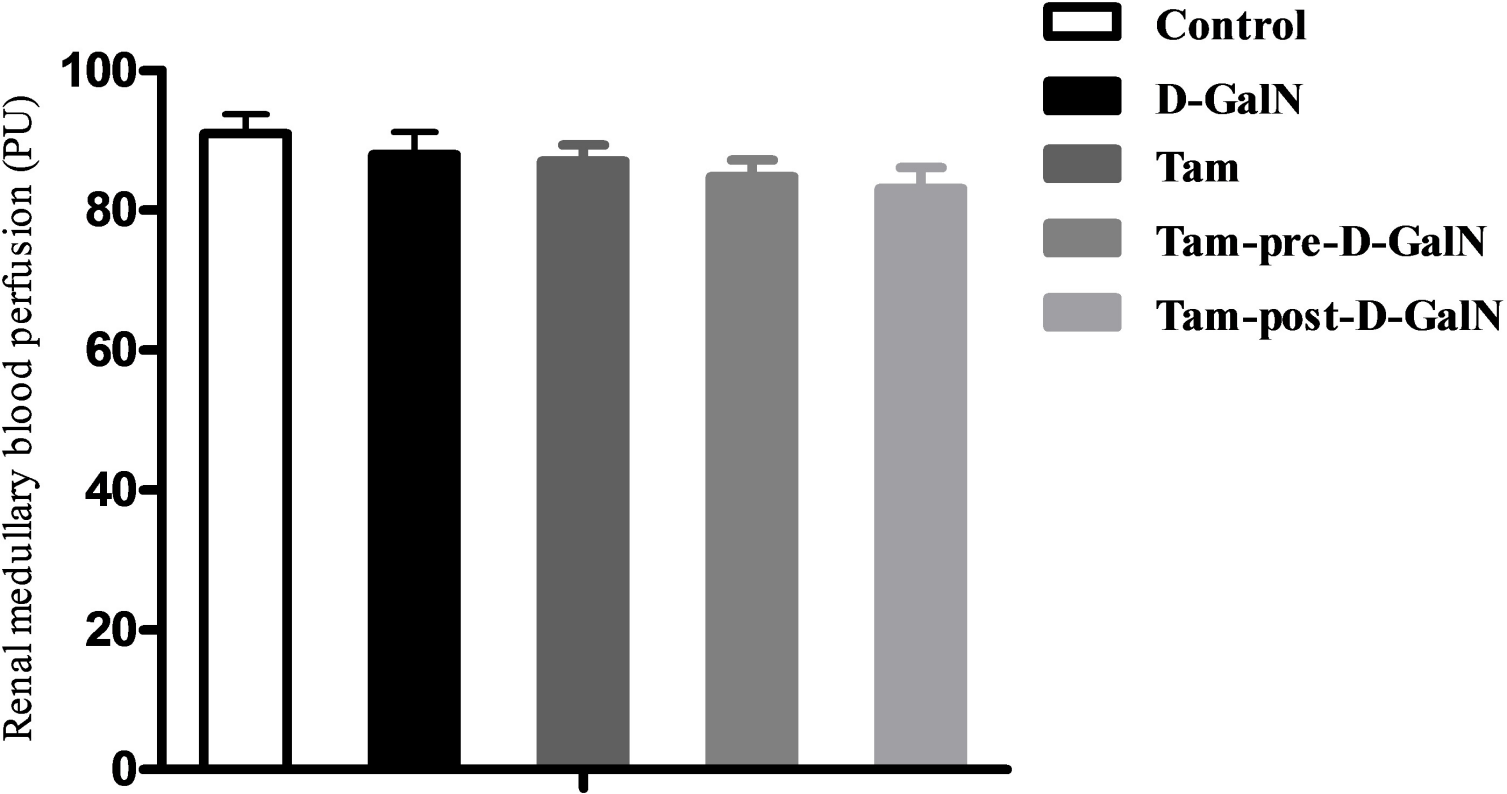
Assessment of renal medullary laser-Doppler flux (MLDF) in D-GalN rats and control rats Control: MLDF were measured at 48 hours after injection of saline. D-GalN: MLDF were measured at 48 hours after injection of D-GalN. Tam: MLDF were measured at 48 hours after administration of tamsulosin. Tam-pre-D-GalN: animals received tamsulosin 72 hours prior to D-GalN intraperitoneally, and MLDF were measured at 48 hours after administration of D-GalN. Tam-post-D-GalN: animals received tamsulosin 36 hours after D-GalN intraperitoneally, and MLDF were measured at 48 hours after administration of D-GalN.

Under baseline condition, the average value of renal cortical LDF (CLDF) was 438.29 ± 41.70 PU in control rats. D-GalN administration resulted in a CLDF of 241.46 ± 14.96 PU, with a decline of 45% from the control group (Figure 5), and a significant difference between control and HRS rats was observed (P < 0.01). The baseline value of renal medullary LDF (MLDF) was 90.97 ± 8.68 PU in Group 1. Forty-eight hours after D-GalN administration, MLDF decreased to 87.93 ± 10.34 PU (5% decline), which did not represent a significant difference between Groups 1 and 2 (Figure 6).

In a separate series of experiments, tamsulosin was (pre or post) given to control rats and rats with D-GalN-induced HRS, and renal blood perfusion (CLDF + MLDF) was monitored as previously described. There were no significant differences in CLDF and MLDF between Group 1 and Group 3, indicating that renal baseline hemodynamic variables were not significantly altered by tamsulosin treatment alone. However, the tamsulosin pre-treatment (Group 4) blunted the CLDF value (328.23 ± 17.67 PU vs. 241.46 ± 16.96 PU; P < 0.01), but it did not affect MLDF (84.82 ± 7.51 PU vs. 87.04 ± 7.31 PU), compared with Group 2 (D-GalN). In contrast, injection with tamsulosin at 36 hours post administration of D-GalN (Group 5) had no effect on either CLDF or MLDF, compared with injection with D-GalN only (Group 2).

### TNF-α level in plasma

**Figure 7:**
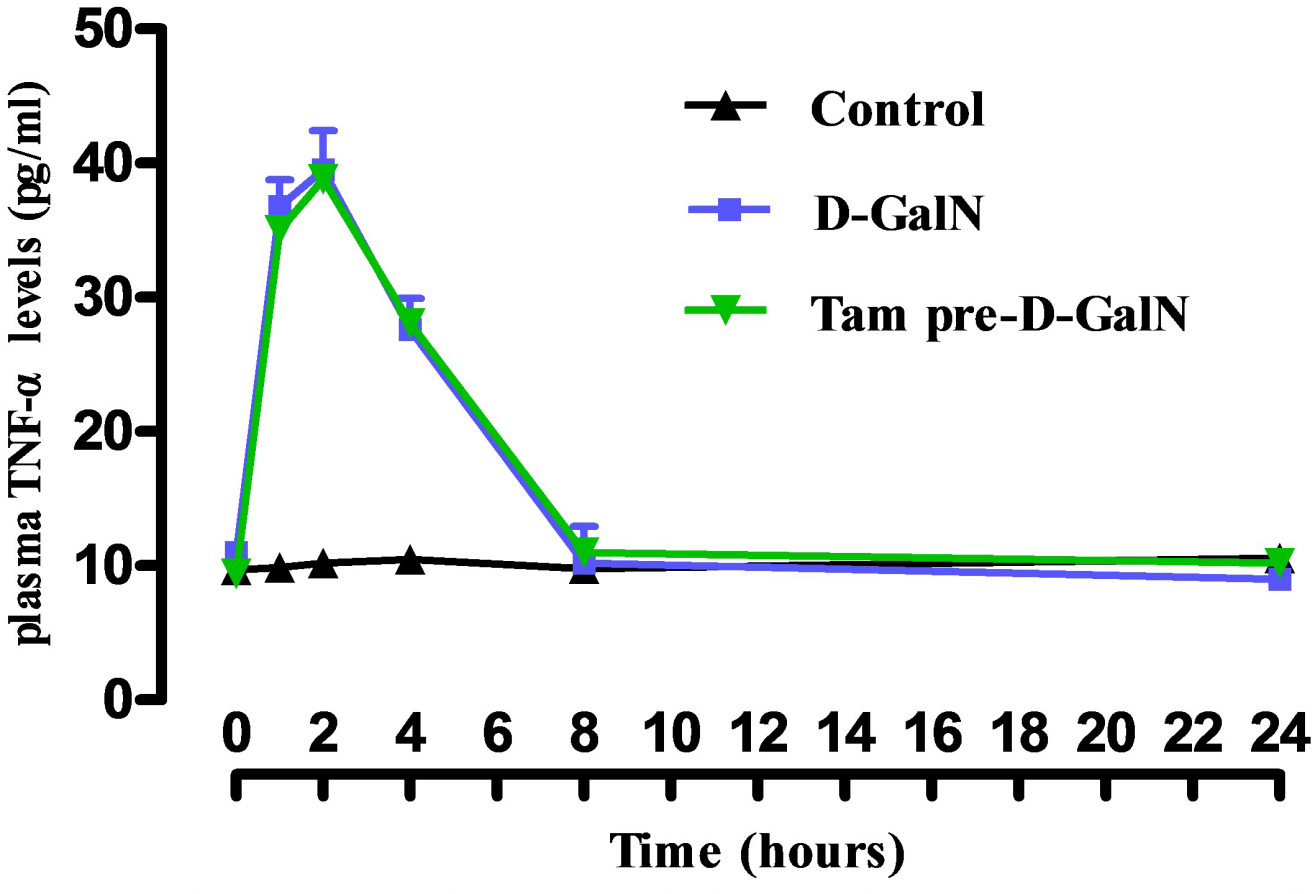
Effects of prior administration of tamsulosin on TNF-α in serum of rats treated with D-GalN Control: received 2 ml of saline intraperitoneally. D-GalN: were administered 1200 mg/kg D-GalN intraperitoneally. Tam-pre-D-GalN: received tamsulosin 72 hours prior to D-GalN intraperitoneally. Data were shown as means ± standard deviations.

TNF-α serum concentration was shown in Figure 7, which was significantly higher in the group that received D-GalN only (Group 2) than in the control group (Group 1). TNF-α increased 1 hour after administration, increased further at 2 hours, and remained elevated for 24 hours compared with the sham group. Changes in serum concentration of TNF-α in tamsulosin-treated animals were undetectable (data not shown). In addition, plasma TNF-α levels in D-GalN only (Group 2) rats were not significantly different from rats received tamsulosin pretreatment (Group 4; P > 0.05).

### Expression of alpha 1 adrenergic receptor

**Figure 8:**
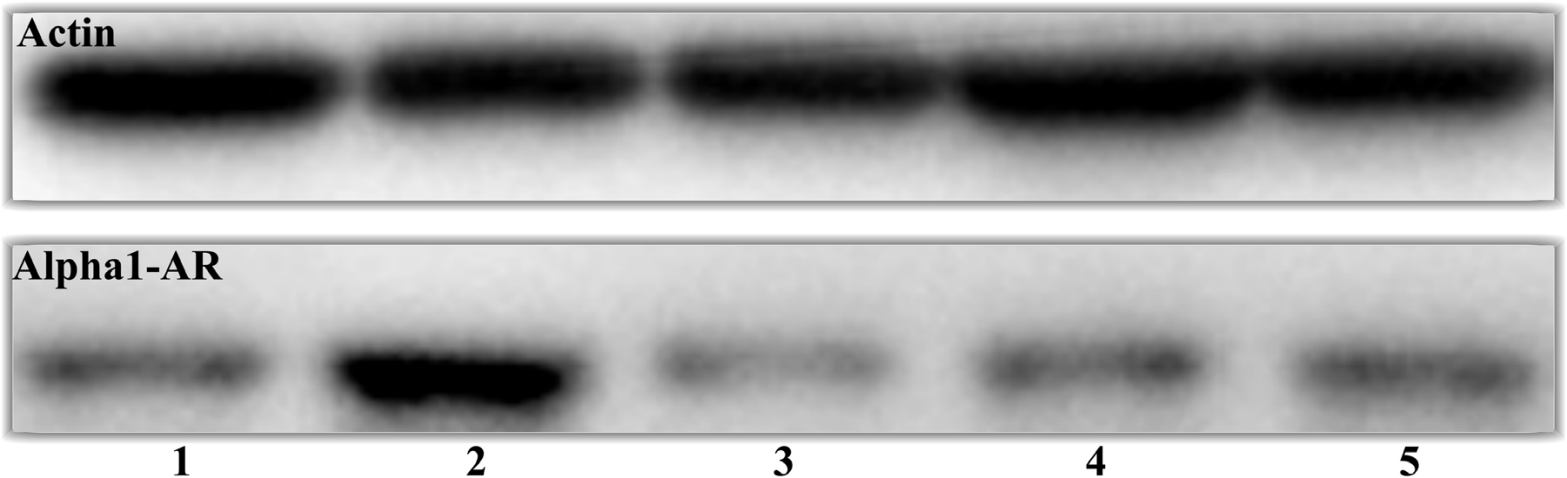
Effect of tamsulosin on α1-AR in HRS rat renal artery Group 1: control (n = 10); Group 2: D-GalN (n = 10); Group 3: tamsulosin (n = 10); Group 4: tamsulosin pre-D-GalN (n = 10); Group 5: tamsulosin post-D-GalN (n = 10). Renal artery tissue was lysed and subjected to immunoblot analysis for anti-α1-AR. Actin was used as control for protein loading.

**Figure 9:**
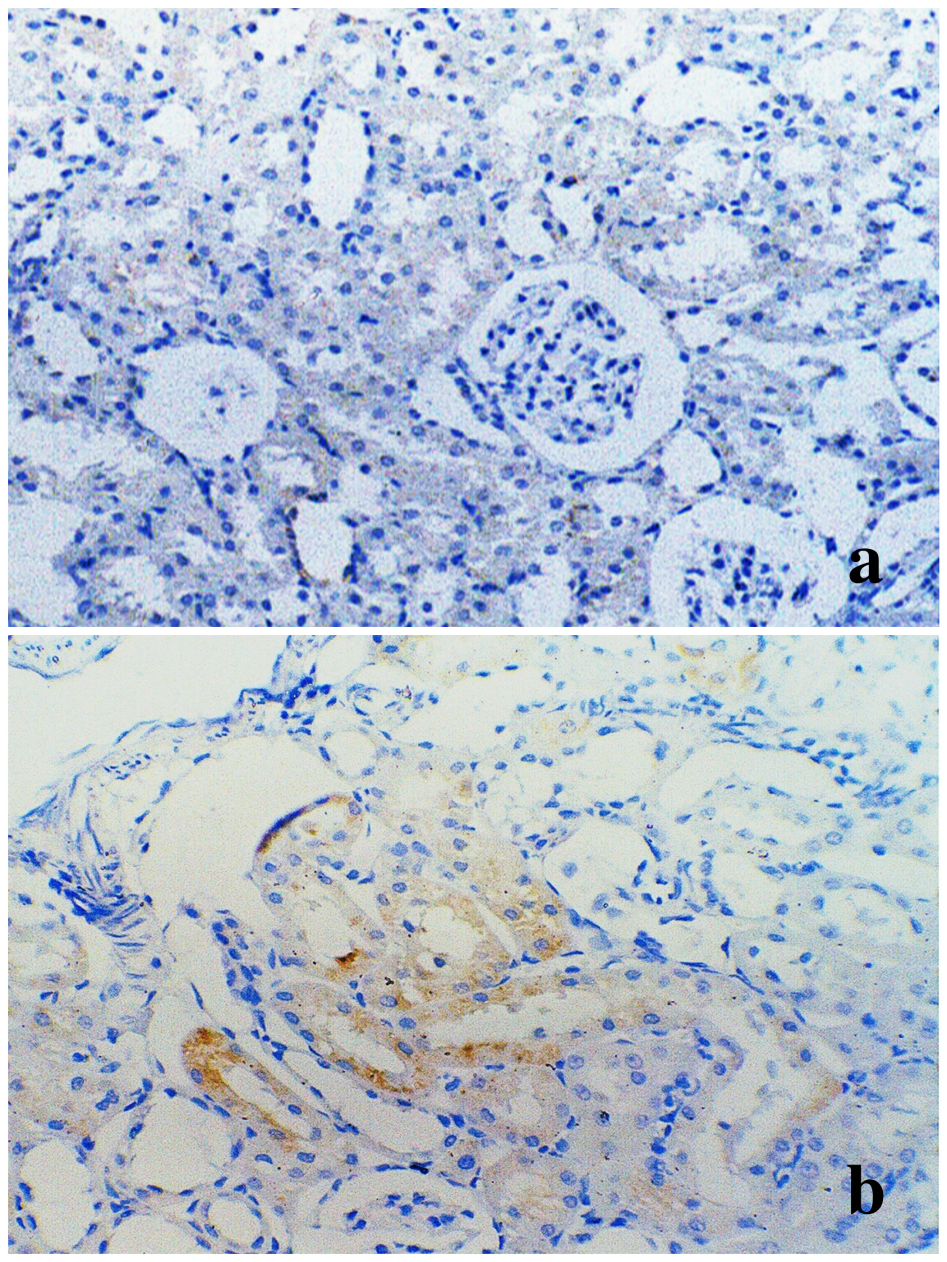
Immunohistochemistry of α1-AR in kidney from control and HRS rats **(a)** Negative immunostaining in renal cortex from control rats (Group 1) (original magnification × 200); **(b)** α1-AR immunoreactivity is observed in proximal tubules from HRS rats (Group 2) (original magnification × 400).

Western blot was used to evaluate α1-AR expression in different treatment groups, compared with control rats (Figure 8). Forty-eight hours after stimulation with D-GalN, the α1-AR protein level was decreased in the tamsulosin pre-D-GalN group (Group 4) relative to the D-GalN only group (Group 2). Similarly, a decline in α1-AR expression was also observed in the tamsulosin post-treatment group (Group 5) compared with Group 2. However, no difference in α1-AR protein expression was observed between the two combination treatment groups (Group 4 and Group 5). These results also suggested that α1-AR expression in the renal artery was increased by ALF induction. In addition, from the immunohistochemistry study, the immunostaining of α1-AR was negative in the kidney from control group, while it was positive in the HRS rats (Figure 9).

## DISCUSSION

A few studies have explored the pathologic mechanisms of renal hypoperfusion in HRS. In our HRS rat model, typical characteristics of ALF, including hepatocellular injury, necrosis of hepatocytes, and inflammatory reaction, were observed. A significant higher TNF-α plasma level, the concentration of which is known to be a predictor of HRS after ALF, was observed 1 hour and 24 hours after administration of D-GalN in our study [10–12]. D-GalN was demonstrated to induce the infiltration of leukocytes and liver cell injury, further releasing proinflammatory cytokines [13, 14]. These cytokines possibly aggravated kidney and renal blood vessel injury, and promoted asystemic inflammatory in response to renal vessel injury [10, 15]. Therefore, the ALF-induced renal injury by D-GalN was probably due to the excessive inflammatory responses in the HRS model [10].

The biochemical events leading to hepatocellular necrosis and liver dysfunction following administration of D-GalN have been investigated in depth over the years, but the characterization and pathogenesis of renal injury and dysfunction have rarely been explored [16, 17]. In our study, interestingly, despite the development of renal dysfunction, the kidney structure was histologically normal. The reduction of UNa in D-GalN-treated rats with HRS implied that tubular function was preserved; however, this may have been influenced by the decreased food and water intake of animals with liver failure. A significant reduction of CLDF and MLDF in kidneys in our model confirmed that renal hypoperfusion occurred independently of any changes in hyperdynamic circulation, and was secondary to renal vasoconstriction. It occurred despite an increase in cardiac output and systemic vasodilatation, as observed in other studies [6, 18, 19]. These major findings indicated that administration of D-GalN to Sprague–Dawley rats caused significant hepatocellular injury, inflammatory response, hyperdynamic circulation, and renal vasoconstriction, which resulted in acute renal failure without irreversible structural renal damage [13, 17]. A previous study demonstrated that D-GalN had no direct toxic effect on cultured LLCPK1 cells, a renal tubular cell line [10, 20]. These results suggested that local changes in pathophysiology, such as renal vascular response and peripheral vascular dilatation resulting from renal vasoconstriction, rather than from the toxic effect of D-GalN, seemed to contribute to HRS as a result of renal hypoperfusion and receptor dysregulation.

HRS is commonly defined as a purely hemodynamic consequence of liver disease resulting in renal failure [5]. In the settings of decreased peripheral vascular resistance, hypovolemia, and the activation of compensatory mechanisms, the kidneys receive even less blood flow resulting in injury. However, Transplant follow-up data consistently shows that pre-transplant renal dysfunction, regardless of acuity, is associated with increased long term renal and overall survivals [11]. These findings are unexpected if HRS is solely hemodynamic and only a manifestation of the degree of systemic circulatory disorder. Therefore, based on our data, we propose that the renal hypoperfusion in HRS is caused by renovascular hyperresponsiveness via α1-AR modulation.

Autoradiographic analyses with specific radio-ligands demonstrated that the renal α1-ARs were mainly located in the cortex, especially in the proximal tubules, with a lower density in the medulla, playing an important role in controlling renal blood flow and electrolytes [21–24]. The effects of ALF on the functional contribution of α1-AR-mediated vasoconstriction of resistance vessels in HRS rats have not been fully elucidated. We found that renal arterial hyperresponsiveness was a key factor, which, even if it did not reach shock values, caused simultaneous renal vasoconstriction and renal hypoperfusion with decreased glomerular filtration. In an attempt to identify possible mechanisms by which renal arterial hyperresponsiveness might cause acute renal ischemia, injury, and dysfunction, we used *in vitro* isometric tension recording on renal artery rings in the HRS rat model. Compared with controls, a higher E_max_ was noted with α1-AR in our HRS rat model, with data showing a highly significant increase in renal vascular response during the 48-hour experimental period after ALF induction. Furthermore, administration of the α1-AR inhibitor, tamsulosin, significantly suppressed hyperresponsiveness of the renal artery contraction, while administration of tamsulosin post-D-GalN did not affect results, suggesting that tamsulosin did not influence the progress of ALF.

Further evidence supported the effectiveness of for α1-AR in the pathogenesis that pre- or post-ischemic treatment with prazosin ameliorated renal dysfunction and tissue injury in ischemia/reperfusion-induced acute renal failure rats [9, 25]. However, no further studies using highly specific AR receptor antagonists have been carried out in the HRS model or in human HRS, and the role of selective AR antagonists in the treatment of HRS is still conjectural. In our study, a decrease in CLDF value and significant upregulation of the AR receptor in the renal cortex were observed in HRS rats. Interestingly, administration of tamsulosin 72 hours before D-GalN reduced the contribution of AR subtype in mediating adrenergically induced renal vasoconstriction and hypoperfusion. However, no difference in MLDF was detected between HRS rats and pre-/post-tamsulosin-treated HRS rats, indicating that neurotransmitters, rather than noradrenaline, may contribute to ALF-induced reductions in MLDF. Previous studies have also found the effect of prazosin on MLDF responses to AR was more difficult to detect than the effect on CLDF responses, simply reflecting the greater variability of receptors in the medulla compared with the cortex [26]. These data, together with findings that tamsulosin prevents the development of renal cortical ischemia, confirmed that AR played an important role as a determinant of renal perfusion in this HRS model.

In summary, α1-AR-mediated hyperresponsiveness of the renal artery contraction give rise to renal vasoconstriction in HRS rats, which may be a mechanism for HRS. Administration of selective adrenergic agonists altered the upregulation of AR subtypes in mediating adrenergically induced renal vasoconstriction and hypoperfusion.

## MATERIALS AND METHODS

### Experimental animals

The experimental protocol was approved by the local ethical committee for animal research of Xi’an Jiaotong University. Male Sprague-Dawley rats (each weighs around 200-250 g) were obtained from Xi’an Jiaotong Comparative Biology Unit, and kept a light-controlled room with a 12 hour light/dark cycle, at a temperature of 19-25°C, and humidity of approximately 55% ± 5%. Rats were placed individually in polycarbonate metabolic cages with free access to food and water. D-GalN was administered as an intraperitoneal injection of 1200 mg/kg (5.5 mmol/kg) in a 200 mg/mL solution in saline (pH 6.8). Controls received 2 mL of saline intraperitoneally. Tamsulosin, a selective α1-AR antagonist was administered daily at 0.03 mg/kg in 1% sodium carboxymethylcellulose.

The animals were divided into five groups by different treatments: Group 1 (control group, n = 10) was given 2 mL of saline intraperitoneally; Group 2 (D-GalN group, n = 10) was administered 1200 mg/kg D-GalN intraperitoneally; Group 3 (tamsulosin controls, n = 10) received tamsulosin orally and saline intraperitoneally; Group 4 (tamsulosin pre-D-GalN, n = 10) received tamsulosin orally 72 hours prior to D-GalN intraperitoneally; and Group 5 (tamsulosin post-D-GalN, n = 10) received tamsulosin 36 hours after D-GalN.

### Biochemical studies

Blood was collected from the caudal vein or inferior vena cava and put into the ethylenediamine tetra-acetic acid or plain tubes and centrifuged at 2000 g for 10 minutes at 4°C. With plasma or serum, it was then stored at -80°C. Quantitative liver and renal function tests, including measurement of levels of aspartate transaminase (AST) and alanine transaminase (ALT), albumin and total bilirubin (TBIL), as well as serum and urine creatinine, and blood urine nitrogen (BUN), were carried out using an automatic analyzer (Hitachi, Japan). Urine sodium concentration (UNa) and plasma sodium concentration (PNa) were determined using a flame photometer (Hitachi, Japan). Creatinine clearance (Ccr) was calculated from urine and serum creatinine values. Fractional excretion of sodium (FENa, %) was calculated using the formula: FENa = UNaV/(PNa × Ccr) × 100, where V is 24-hour urine volume.

### Vascular response to stimulation with vasoconstrictor

The renal artery was immediately removed and carefully cleaned of connective tissue and blood, then cut into 2 or 3 mm renal arterial rings. These rings were mounted on two L-shaped stainless steel pins in a myograph chamber. One pin was connected to a force displacement transducer (JH-2) to record isometric tension with a MultiMyograph System-610M (Danish Myo Technology A/S, Denmark) attached to a digital converter unit. Another pin was connected to a manual screw, which allowed for fine vascular tone adjustments by varying the distance between the two pins. Measurements were recorded on a computer using a Power Lab Unit (AD Instruments, Oxford, UK). The renal arterial rings were immersed in a temperature-controlled buffer solution (37°C), which was continuously gassed with a 5% CO_2_ in O_2_ gas mixture, resulting in a stable pH of 7.4. During an equilibration period of 120 minutes, a baseline tension of 2 mN was adjusted. After 2 hours of equilibration, potassium-rich (60 mM) Kreb’s buffer solution was used to determine segment contractile function as a contractile capacity reference. When two reproducible contractions had been achieved, the vessels were used for further experiments. Vascular segment concentration-response curves were obtained by cumulative administration of NE (10-8-10-4.5 mM). E_max_ represented the maximal constriction induced by NE (1 × 10^-10^–1 × 10^-3^ mol/L) in preconstricted arterial rings. PD2 represented the negative logarithm of the concentration that produced 50% of the maximal contractile effect.

### Cortical and medullary perfusion measurements

Anesthesia was induced with 5% isoflurane followed by 2.5% in 30% oxygen–70% nitrous oxide. After 48 hours of D-GalN or saline administration, the left kidney was exposed by midline abdominal incision. Renal cortical and medullary perfusion were measured with a Perimed FeriFlux System 5000 flowmeter (Sweden), using a microprobe secured with a self-adhesive probe (which reduces the impact of artefactual movement and ensures good optical coupling) applied to a point on the upper left anterior surface of the kidney. The laser-Doppler flux (LDF) signal from the kidney, corresponding to the cortical perfusion, was sampled at 100 Hz and measured using a time constant of 0.03 seconds.

### Histology, immunohistochemistry, and western blotting

In the end, rats were euthanized by stunning and exsanguination. Liver and kidney samples were fixed in 10% neutral buffered formalin, embedded in paraffin, sectioned, and stained with hematoxylin and eosin for light microscopy examination. To study the vascular microstructure, a transmission electron microscope (TEM, H-600, Hitachi, Japan) was used. The fresh vessels were sectioned at 1 mm × 3 mm × 3 mm and fixed with 2.5% glutaraldehyde + 4% paraformaldehyde in phosphate buffer for 2 hours at 4°C, and washed with 0.1 M phosphate buffer for 30 minutes. Ultrathin sectioning at 50-70 nm was carried out (LKB-V, Sweden); sections were stained with uranyl acetate and lead hydroxide for 10 minutes, and subsequently examined in the transmission electron microscope.

After homogenizing renal artery tissue, total protein was extracted. A Coomassie brilliant blue assay was used to determine total protein concentration. Proteins were resolved by 10% sodium dodecyl sulfate polyacrylamidegel, transferred to a nitrocellulose filter membrane using a semidry electrophoretic graphite electrode with a constant current of 1 mA/cm^2^ gel for 1 hour, and probed with the diluted specific antibody to α1-AR (β-actin was used as an internal standard), followed by horseradish-peroxidase-conjugated secondary antibody. An antibody chromogenic agent (Santa Cruz Biotechnology Inc, USA) was placed on the membrane for the development of a film, and a scanner was used for imaging. Densitometry was used to measure the protein level.

### Statistics

All data were expressed as mean values ± standard error of the mean. The concentration-effect curves of agonists were fitted to the Hill equation using an iterative, least square method (Graph Pad Prism, San Diego, CA, USA) to provide estimates of maximal contraction (E_max_) and pEC50 values (negative logarithm of the concentration that produced 50% of the maximal effect). Two-way analysis of variance (ANOVA) with Dunnett's test post-hoc was used for comparisons between all treatment groups. P < 0.05 was considered as statistically significant. The comparison of histology scores was analyzed by the Mann-Whitney test.
